# Classification of chikungunya cases: a proposal

**DOI:** 10.1590/0037-8682-0529-2020

**Published:** 2020-11-25

**Authors:** Carlos Alexandre Antunes de Brito, André Ricardo Ribas Freitas, Rodrigo Fabiano Said, Melissa Barreto Falcão, Rivaldo Venâncio da Cunha, André Machado Siqueira, Maria Glória Teixeira, Guilherme Sousa Ribeiro, Marina Coelho Moraes de Brito, Luciano Pamplona de Góes Cavalcanti

**Affiliations:** 1 Universidade Federal de Pernambuco, Departamento de Medicina Clínica, Recife, PE, Brasil.; 2 Instituto de Pesquisa Autoimune, Recife, PE, Brasil.; 3 Ministério da Saúde do Brasil, Comitê Técnico de Arboviroses, Brasília, DF, Brasil.; 4 Faculdade São Leopoldo Mandic, Campinas, SP, Brasil.; 5 Organização Panamericana de Saúde, Brasília, DF, Brasil.; 6 Universidade Estadual de Feira de Santana, Núcleo de Pesquisa e Extensão em Vigilância à Saúde, Feira de Santana, BA, Brasil.; 7 Fundação Oswaldo Cruz, Campo Grande, MS, Brasil.; 8 Universidade Federal do Mato Grosso do Sul, Escola de Medicina, Campo Grande, MS, Brasil.; 9 Fundação Oswaldo Cruz, Instituto Nacional de Infectologia Evandro Chagas, Rio de Janeiro, RJ, Brasil.; 10 Universidade Federal da Bahia, Instituto de Saúde Coletiva, Salvador, BA, Brasil.; 11 Universidade Federal da Bahia, Escola de Medicina, Salvador, BA, Brasil.; 12 Fundação Oswaldo Cruz, Instituto Gonçalo Moniz, Salvador, BA, Brasil.; 13 Universidade Federal de Pernambuco, Centro de Ciências Médicas, Recife, PE, Brasil.; 14 Universidade Federal do Ceará, Escola de Medicina, Departamento de Saúde Comunitária, Fortaleza, CE, Brasil.

## INTRODUCTION

Since 2005, chikungunya virus (CHIKV) has caused large outbreaks, with reports of more than 10 million cases worldwide over the course of a decade^1^. Although musculoskeletal involvement is the most prevalent symptom in the disease spectrum, systemic manifestations with involvement of important organs have also been described^2^ ^-^ ^4^.

Currently, case classifications of chikungunya based on the intensity and duration of articular symptoms have been used to guide the clinical management of the joint manifestations, with little or no emphasis on the severe clinical forms associated with systemic disease and target organ involvement. Thus, these classifications have failed to aid health professionals in the detection and early management of severe cases, which can lead to fatal outcomes and sequelae^5^.

A group of experts decided to review the classification of chikungunya fever cases to raise awareness to the severe forms of chikungunya with the aim of reducing mortality, which is aligned with the World Health Organization (WHO)’s goals of decreasing fatal outcomes of vector-borne diseases by 50% and 75% by 2025 and 2030, respectively. The proposed classification system will be incorporated into the new guidelines for the diagnosis and treatment of chikungunya fever of the Brazilian Ministry of Health, which are already under development.

The findings from chikungunya clinical studies and the accumulated experience of specialists during epidemics in India, the Caribbean, and, most recently, Brazil support the need for adjustments to the current classifications^6^. We believe that a revised classification system should be based on an expanded description of the different clinical presentations of the disease, expressing the various biological gradients, which should range from mild to severe, as would be expected for an infectious disease (Evans’ Fifth Postulate). A classification system encompassing the full disease spectrum will help increase health professionals’ awareness of the severity and complications associated with each category, similar to the systems that have been developed for countless other epidemic infectious diseases. A structured classification system allows the standardization of treatment according to clinical complexity and severity, guiding actions such as the definition of the treatment setting based on disease complexity, ordering of complementary exams, and initiation of therapies.

## DISCUSSION

In 2015, a panel of experts revised the classification of chikungunya cases and proposed four disease categories: acute, atypical, severe acute, and chronic^7^. Although this proposed classification system represents an important improvement, it still has limitations, as the main symptom used to categorize the patients is joint impairment, without focusing on systemic symptoms that can occur. A study performed in a university hospital in Martinique showed that the current chikungunya fever classification system produced by the WHO is especially ineffective for elderly patients. Of the 257 patients older than 65 years with laboratory confirmation of CHIKV infection with positive reverse transcriptase polymerase chain reaction results, 42.7% could not be classified in any category of the current system, and in the group of 109 younger patients (<65 years), 17.4% could not be classified, showing that this model has limitations. Additionally, in the group aged 65 years and older, only 8.2% were classified as having the typical form as defined by the WHO^8^.

Furthermore, the pathophysiological mechanisms of the main arbovirus infections, including chikungunya, reinforce the concept that the disease is dynamic and that the impairment of systemic organs (classified as atypical forms) can evolve into severe forms and become the main cause of death related to CHIKV infections. Therefore, at any stage, systemic manifestations should represent an important sign of severity. The atypical and acute severe groups suggested in the 2015 modification of the classification system represent the same group with different biological levels of intensity.

Several of the terms used in the classification system may need to be revised in the process of creating a categorization system that can ultimately enable professionals to detect cases. The term “atypical” refers to that which does not correspond to the predominant clinical features of chikungunya but rather to a situation that is unexpected, which minimizes the relevance; however, it is clear that the impairment of systemic organs is part of the spectrum of the disease, even if it is less common. Similar patterns have been observed in numerous other epidemic viral diseases (dengue, yellow fever, and influenza).

### Expanded clinical spectrum of the disease

Musculoskeletal manifestations are the most common, and their management is related to different phases^9^ ^-^ ^10^. Chronologically, the spectrum of the disease involves three phases (acute, postacute, and chronic). Clinically, there are different patterns of joint manifestations that may be predominantly noninflammatory/musculoskeletal (mechanical) or inflammatory with arthritis and periarticular manifestations. Approximately 50% of patients develop chronic joint disease, with important impairment of their quality of life, and, in many cases, mimicking clinical patterns of rheumatic diseases such as rheumatoid arthritis (RA), seronegative spondylarthritis (SpA), fibromyalgia (FM), and undifferentiated polyarthritis^10^ ^-^ ^13^. 

In addition to musculoskeletal involvement, there are relevant systemic manifestations with the involvement of important organs. Systemic manifestations that can evolve with exacerbation mainly affect the nervous, cardiac, pulmonary, renal, hepatic, endocrine, and vascular systems^2^ ^-^ ^4^.

In 2016 on Reunion Island, among the 123 cases of severe chikungunya fever, the main reasons for hospitalization were respiratory failure (19 cases), cardiovascular decompensation (18), meningoencephalitis (16), severe hepatitis (11), severe skin lesions (10), and renal failure (7)^4^.

Among the 96 nonpregnant adults enrolled in a study by Bonifay et al. in French Guiana during the 2014/2015 CHIKV outbreak, 29% of the patients were classified as having atypical or severe forms, with the most common symptoms being neurological symptoms (Guillain-Barret syndrome, encephalitis, seizure, confusion, and stroke), followed by cardiorespiratory failure, hepatitis, pancreatitis, renal failure, and muscular impairment^2^.

Severe forms of the disease and fatal outcomes have been described as more often affecting those at the extreme ends of the age spectrum, predominantly elderly patients, and the presence of comorbidities may contribute to the development of complications^4^. However, severe forms have also been described in different age groups in the absence of associated diseases^14^ ^-^ ^16^. In a study of 44 patients with arrhythmia conducted by Economopoulo et al., 63% had no history of cardiovascular disease, and in 131 patients with blood glucose level alterations, 20% were diagnosed with diabetes mellitus for the first time^3^.

The signs and symptoms described in the systemic form of chikungunya fever are related to the injury of target organs by the disease^17^ ^-^ ^18^. In a systematic review of the systemic manifestations associated with chikungunya, cardiac involvement was reported in 54% of the articles, and the predominant symptoms were chest pain, fatigue, dyspnea, palpitations, vagal symptoms (diaphoresis, pallor, and coughing), nausea, dizziness, and lipothymia/syncope. On physical examination, the findings included tachycardia, atrial and ventricular premature ectopic beats, crepitation or rhonchi in the lungs, and tachypnea^17^.

Mehta R et al., in a systematic review of neurological impairment due to chikungunya, found encephalitis to be the most common complication, followed by myelopathy, myelitis, and Guillain‐Barré syndrome^18^. The most common symptoms included headaches, altered sensorium, lethargy, seizures, weakness, and paresthesia^18^.

Studies conducted first in India and more recently in Brazil reported significantly higher mortality rates in the months coinciding with chikungunya outbreaks than in the same period in previous years and the expected rates after the outbreak. Retrospective analyses in different Latin American countries have produced similar results, demonstrating a strong temporal association and potential causality between this surplus mortality and the outbreak^19^ ^,^ ^20^.

It is possible that the failure to accurately identify the mortality rate associated with chikungunya is related not only to flaws in the surveillance system but also, and perhaps mainly, to the lack of an awareness of the possibility of CHIKV infection as a cause death on the part of doctors. In part, this can be justified by the limited knowledge of serious systemic manifestations and possible complications related to CHIKV infection and the lack of a case classification system that contributes to increasing the awareness of health professionals about the spectrum of this disease.

### RESULTS: A structured classification system based on the clinical spectrum of the disease

**FIGURE 1:**
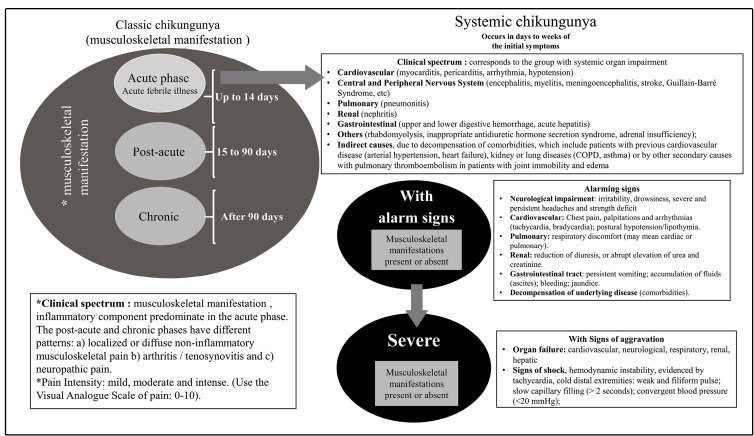
Proposal of new classification system for chikungunya cases.

The clinical spectrum of the disease chronologically involves three phases: an acute phase (up to 14 days), a postacute phase (15 to 90 days), and a chronic phase (after 3 months). The disease should be classified according to the clinical spectrum and level of intensity as follows:

- “Classic chikungunya”, divided into 3 phases according to the duration of symptoms: “acute”, “postacute”, and “chronic”; and

- “Systemic chikungunya”, divided by severity into “chikungunya with alarm signs” and “severe chikungunya” (Figure 1).

1. Classic chikungunya: The classic form of chikungunya is characterized by musculoskeletal impairment, which is the most common clinical manifestation of the disease with an inflammatory component predominantly in the acute phase. The postacute and chronic phases have different patterns of manifestations that can involve: (a) localized or diffuse noninflammatory musculoskeletal pain; (b) arthritis/tenosynovitis (articular or periarticular disease associated with edema); and (c) neuropathic pain. It is important to emphasize that a patient can present with a combination of patterns such as arthritis or noninflammatory musculoskeletal pain associated with neuropathic pain.

2. Systemic chikungunya: These forms are characterized by systemic manifestations resulting from organ impairment that may or may not present simultaneously with or be preceded by musculoskeletal manifestations. The clinical spectrum involves systemic organ impairment due to the direct effects of the virus, systemic inflammatory response, or indirect causes (as a consequence of decompensating underlying disease). The systemic impairment of organs can involve the following: cardiovascular (myocarditis, pericarditis, arrhythmia, and hemodynamic instability); central and peripheral nervous system (encephalitis, myelitis, meningoencephalitis, stroke, Guillain-Barré syndrome, optic neuritis, cerebellitis, and rhombencephalitis); pulmonary (pneumonitis); renal (nephritis); gastrointestinal (upper- and lower-digestive hemorrhage, and acute hepatitis); others (rhabdomyolysis, inappropriate antidiuretic hormone secretion syndrome, and adrenal insufficiency); indirect causes due to decompensation of comorbidities, including preexisting cardiovascular (arterial hypertension and heart failure), kidney, or lung (COPD and asthma) diseases, or other secondary causes like pulmonary thromboembolism in patients with joint immobility and edema (Figure 1).

With regard to chronology, these manifestations may occur days or weeks after the initial symptoms appear. The timing of the event depends on the causes and organs affected, while the gradient of intensity is classified as chikungunya with alarm signs or severe chikungunya, which can be identified by the presence of alarm signs or their aggravation, respectively (Figure 1). Alarm signs manifest when target organs are affected. The presence of alarm signs should be defined as a separate group, since it indicates the presence of clinical conditions that can progress with aggravation and carries the risk of a fatal outcome, necessitating careful medical care and specialized treatment.

In the severe form**,** the patient experiences aggravation of the clinical features, with worsening of alarm signs due to organ failure that may be cardiovascular, neurological, respiratory, renal, and/or hepatic. Signs of shock represent one of the manifestations of aggravation related to systemic cardiovascular impairment.

## CONCLUSION

We believe that the proposed classification system takes into consideration the different clinical manifestations of CHIKV infection, thus facilitating the construction of flowcharts and guiding the development of future clinical guidelines for addressing the complete spectrum of the disease and increasing awareness of this spectrum, enabling health care professionals to identify and treat systemic chikungunya before the development of worse outcomes.
